# Instrument-Free Point-of-Care Diagnostic for Leishmania Parasites

**DOI:** 10.3390/diagnostics14232744

**Published:** 2024-12-05

**Authors:** Taralyn J. Wiggins, Ruonan Peng, Ruth V. Bushnell, John K. Tobin, David A. MacLeod, Ke Du, Gregory J. Tobin, Stephen J. Dollery

**Affiliations:** 1 Biological Mimetics, Inc., 124 Byte Drive, Frederick, MD 21702, USAtobin@bmi-md.com (G.J.T.);; 2 Chemical and Environmental Engineering Department, University of California at Riverside, B350 Bourns Hall, 900 University Ave, Riverside, CA 92521, USA

**Keywords:** leishmaniasis, neglected tropical disease, point-of-care diagnostics, instrument-free

## Abstract

Background/Objective: Leishmaniasis is the second deadliest parasitic disease in the world, after malaria, with an estimated 1.6 million new cases each year. While cutaneous leishmaniasis can result in permanent scars from lesions after treatment, the mucocutaneous and visceral diseases can result in life-altering and life-threatening complications. Accurate species diagnosis is critical for treatment and follow-up, and while PCR-based diagnostics can provide sensitive parasite detection and species identification, they are slow, expensive, and not suitable for low-resource settings. In this publication, we describe our efforts to develop a simple, affordable, and instrument-free Leishmania DNA diagnostic that can be used in both high-tech settings and the field. Methods: Computational biology was utilized to design region-targeted RPA oligos and the corresponding CRISPR guides for the detection of all Leishmania species as well as the specific identification of *L. (V.) panamensis* as a predictor of mucocutaneous disease. Then, we executed systematic approaches for parasite lysis, RPA amplification of DNA, and fluorescent CRISPR crRNA detection. Results: We have demonstrated the ability to detect single-digit parasites without compromising the specificity in identifying single species as the proof of concept for a point-of-care diagnostic. Individual assays were carried out in succession, culminating in an unquenched fluorescent signal quantifiable over negative control. Conclusions: The described work is the foundation which will be implemented into a three-track [all Leishmania, mucocutaneous or visceral only, and a human positive control] assay that we plan to utilize in a Funnel Adapted Sensing Tube (FAST) single use, instrument-free, and affordable diagnostic.

## 1. Introduction

Leishmaniasis ranks among the top ten neglected tropical diseases (NTD), with 0.7 to 2 million new cases with 12 million overall prevalence, 20,000 to 30,000 deaths, and >350 million people at risk of infection per year [1,2,3]. These numbers are presumed to be underestimations, due to non-mandatory reporting and unrecognized cases [4]. Leishmania parasites are endemic in almost 100 countries, spanning large areas of the tropics and subtropics which can be divided into “Old World” (Europe, Africa, Asia) and “New World” (North America, South America, Caribbean) based on geographic location [1,5,6]. Individuals infected with Leishmania parasites can suffer from three main forms of disease: visceral (VL or kala-azar, black fever), cutaneous (CL) and mucocutaneous (MCL). Visceral disease, the most serious, impacts several internal organs, such as the spleen and liver, and can be fatal if left untreated [7,8]. A secondary complication of VL is skin rashes consisting of macules, papules, or nodules (post-kala-azar dermal Leishmaniasis) [9]. Cutaneous disease, the most common, causes skin sores which can vary in severity, number and appearance [10]. MCL impacts the mucosal membranes of the nose, mouth and throat, which can lead to permanent disfigurement and pain [11]. In 2020, of the 200 countries and territories that reported to the WHO, 98 (49%) were considered endemic for Leishmaniasis [12]. Both MCL and VL can initially present with cutaneous lesions; therefore, the rapid diagnosis of more severe disease-causing species is vastly important [13,14]. While the majority of the 21 species that infect humans cause CL, *L. (L.) donovani* and *L. (L.) infantum*, are most commonly associated with VL in the Old World, whereas *L. (V.) brazilensis, L. (V.) panamensis*, and *L. (V.) guyanensis* are most commonly associated with MCL in the New World, and *L. (L.) infantum* also caused LV in the New World, especially in Brazil [10,15].

A consensus from clinicians in the endemic regions of low- and middle-income countries (LMIC) indicates that the most accurate and reliable methods of diagnosis, PCR and qPCR, are generally restricted to larger clinics in more urban settings and, where available, can be cost prohibitive at >USD 45 [16,17,18,19]. The initial diagnosis of Leishmaniasis is typically performed using a microscopic examination of lesion scrapings for CL or blood and bone marrow aspirates for VL [20,21,22]. When Leishmaniasis is suspected, nucleic acid-based tests, such as PCR or qPCR, are used for definitive diagnosis. In low-resource settings, confirmative diagnostic tests can be impacted by factors such as the distance to clinic, lack of electricity, availability of supplies, instrumentation and/or trained personnel [17,23]. Samples can be sent to central laboratories, but the results are often not available for two or more weeks, which can make patient follow-up difficult and delay treatment. In addition, the cost of DNA purification and PCR (approximately USD 45 per test) can be a barrier.

What is lacking is an accurate and sensitive, low-cost, instrument-free Leishmania diagnostic that can be deployed into the field and used in low-resource settings [24]. Current available options that are compatible with field detection include clinical evaluations and either microscopic examinations (lesion scrapings, biopsy impression smears and histopathology) or rapid antibody tests including IT Leish from Global Access Diagnostics (Bedford, UK), CL Detect ™ from InBios International, Inc. (Seattle, WA, USA), and Signal®KA from Span Divergent Ltd. (Surat, GJ, India) [25,26,27]. The limitations of microscopic examinations include the inability to distinguish between different species of Leishmania [1,28]. The limitations of antibody tests include the inability to distinguish between active and past infections. False positive diagnoses may lead to unnecessary therapies with expensive and toxic drugs while false negative diagnoses correlate to prolonged disease course and greater patient suffering [28].

The treatment of Leishmaniasis is determined by the type of disease, stemming from parasite identification, and can be involved and expensive, especially for impoverished regions where traveling into city centers is required for PCR confirmation and treatment [29,30]. Although there is no universal treatment procedure for Leishmaniasis, current practices include intravenous pentavalent antimonials, chemotherapies and antifungal azole drugs, which require residing at or near treating hospitals with durations in excess of 28 days [5,31,32]. Due to the frequent side effects of the drugs, aggressive treatment is often reserved for those with visceral, mucocutaneous, or severe cutaneous disease.

To improve the diagnostic process, we are developing a rapid nucleic acid diagnostic that requires no electricity or instrumentation and can be used in the field, point-of-care clinics, or low-resource settings, as well as advanced care and state-of-the-art hospitals [33,34,35,36]. In the final configuration, reagents will be lyophilized and placed into the diagnostic for extended shelf life and thermostability, therefore enabling the diagnostic to not only operate without electricity but be stored without. The process is sensitive and specific, rivaling the results produced by PCR testing in laboratory settings. The technology uses the detergent lysis of parasites to release target DNA, Recombinase Polymerase Amplification (RPA) to boost sensitivity, and finally, Clustered Regularly Interspaced Short Palindromic Repeats (CRISPR-Cas12a) for specific detection. In this report, we present data derived using thin-wall PCR tubes and plan to adapt the assay to a novel multichambered “Funnel-Adapted Sensing Tube” (FAST) device. The diagnostic will incorporate reactions for the identification of (1) all Leishmania species while avoiding the detection of *Trypanasoma* spp. [All], (2) selected species that cause either MC or VL disease [MC], and (3) a final chamber for the detection of human DNA as a positive control [Human Control]. We demonstrate the detection of target DNA sequences from less than one parasite and the differential detection of *L. (V.) braziliensis*, as a species that can cause MCL.

## 2. Materials and Methods

### 2.1. Propagation of Parasites and Use as Targets

The following reagents were obtained through BEI Resources, NIAID, NIH: Leishmania parasite species: *Leishmania (Viannia) braziliensis*, Strain HOM/BR/75/M2903, NR 50608; *Leishmania (Viannia) panamensis*, Strain PSC-1 (MHOM/PA/94/PSC 1), NR-50162; *Leishmania (Leishmania) infantum*, Strain HOM/CN/93/KXG-LIU, NR 50605; *Leishmania (Leishmania) venezuelensis*, Strain MHOM/VE/80/H-16, NR-29184; *Leishmania (Leishmania) tropica*, Strain HOM/TR/99/EP41, NR-51828; *Leishmania (Leishmania) gerbilli*, Strain RHO/CN/62/20, NR-50601; *Leishmania (Leishmania) donovani*, Strain HOM/IN/83/AG83, NR-50602. The parasites were propagated in a tissue culture. Briefly, frozen samples of the parasites were inoculated into T-25 flasks with Modified M199 medium (Gibco (Grand Island, NY, USA; Ref: 12350-039), supplemented with 10% Heat-Inactivated Fetal Bovine Serum [HIFBS] (Neuromonics (Edina, MN, USA); Cat. No. FBS006) and 10 ug/mL Hemin Chloride (Millipore Sigma (Burlington, MA, USA); Ref: 3741-5GM) at 25 °C, then expanded into T-182 flasks (CellTreat (Ayer, MA, USA); Ref:229351). When peak density was achieved, the parasites were cryopreserved in 0.5 mL aliquots at a final concentration of approximately 3 × 10^7^ parasites/mL in fresh medium with 5% DMSO (Fisher (Frederick, MD, USA); Ref:BP231-1) in liquid nitrogen for long-term storage. When used as targets in assay development, the parasites were enumerated using a cytometer, pelleted from growth media at 2000× *g*, and washed with phosphate buffered saline (PBS) (Gibco (Grand Island, NY, USA); Ref:10010-031). For purified DNA samples, the parasites were extracted twice each with phenol-chloroform (1:1) (Fisher (Frederick, MD, USA): Ref:BP1750I-100/BP1145-1) than additionally chloroformed (Fisher (Frederick, MD, USA); Ref: BP1145-1), and then ethanol precipitated (Fisher (Frederick, MD, USA); Ref: A407P-4). For use as crude parasites, the washed parasites were resuspended in PBS for the addition of detergents. During these processes, the concentration of parasites in each sample was recorded.

### 2.2. Parasite Lysis

Initial Lysis: 12 mL of peak density *L. (V.) braziliensis*, or other species, were harvested, pelleted, washed with 10 mL of PBS, pelleted, resuspended in 12 mL of PBS (Gibco (Grand Island, NY, USA); Ref:10010-031) and aliquoted into 1.5 mL micro-centrifuge tubes. The parasites were pelleted and supernatant decanted, then 200 µL of a 0.1% or 0.5% (*v*/*v*) solution of the detergents was added; CHAPS (Fisher (Frederick, MD, USA); Ref: BP571-5), Triton X-100 (Acros (Geel, Belgium); Ref: 21568-2500), NP40 (Boston BioProducts (Milford, MA, USA); Ref: P-872), Tween-20 (Fisher (Frederick, MD, USA); BP337-500), n-octyl glucopyranoside (Affymetrix (Santa Clara, CA, USA); Ref: 29736-26-8) detergents were added and exposed to 56 °C for 10 min. The negative control was an equivalent number of parasites with 1× PBS only. The extent of lysis by each detergent was determined microscopically. The compatibility with enzymatic reactions was determined by using an RPA reaction of 2.75 µL of lysed DNA to an equivalent number of targets in phenol-extracted DNA. As an additional test, 100 µL of each lysis condition were added into a 12-well plate (TrueLine (Greenville, SC, USA); TR5001) with 3 mL of culture media and incubated for 4 days to observe for propagation as a sign of un-lysed parasites.

Master preparations of lysed parasites were stored at −20 °C between experiments.

### 2.3. Recombinase Polymerase Amplification [RPA]

RPA amplification was conducted using the TwistAmp^TM^ Basic Kit (TwistDx (Scarborough, ME, USA) Part #: INTABAS), using a modified protocol of 14 µL reactions in PCR tubes [33,34,35,36,37,38,39]. Each reaction tube consisted of 1 µL each of 5 µM forward and reverse primers, 6 µL of rehydration buffer, 2.5 µL of previously diluted polymerase reaction mix [10 µL nuclease-free water added to the TwistAmpTM reagent tubes], ~2.75 µL Template DNA, 0.8 µL MgOAc, and ~2.45 µL NG water to final volume. Standard reactions were incubated at 39 °C for 20 min. For visualization on 2.5% agarose gels (BioRad (Hercules, CA, USA); Ref: 161-3102), 1 µL of a 0.1 mg/mL solution of BioLab SYBR Green (Biotium (San Francisoco, CA, USA); Ref: 40086) and 2 µL of 6× tracking dye (NEB (Ipswich, MA, USA); B70245) were added to each tube prior to electrophoresis. A variety of temperatures and incubations times were evaluated to determine RPA parameters and detergent compatibilities

RPA primers were designed after identifying primer sites that flank potential CRISPR guide sequences through the analysis of multiple sequence alignments. Kinetoplast maxicircle DNA (kDNA) was targeted for the diagnostic due to each parasite having 30–50 copies. Maxicircle DNA contains a mixture of sequences that are both conserved and variable between Leishmania species. The region targeted for detection maps to nucleotides 1808 to 2769 in the *Leishmania (V.) panamensis* strain PSC1 (maxicircle) kinetoplast (BK010875). Seven forward and eight reverse primers were selected for evaluation and purchased from Integrated DNA Technologies (IDT, Coralville, IA, USA) (Table 1).

### 2.4. CRISPR

For consensus guides, the guide tool CRISPOR (hosted by UC Santa Cruz at crispor.org) was used to score and select potential guide target regions. For less common specific guides, manual selection, or bespoke algorithm aided selection (Python), was performed to screen for guides. The IDT website tools were used for generating the remaining constant RNA using the CRISPR-Cas12a guide oligos design tool. In CRISPOR, Step 1 is to input a created consensus sequence for each of the conserved regions, Step 2 is the selection of the *Homo sapiens* human target genome, and Step 3 is the identification of the [TTT(A/C/G)-23 bp–Cas12a (Cpf1)]–recommended, guides. The highest scoring guides were selected for region 1 (All-1, All-2) and region 2 (All-3, All-4). The target regions for the guides are shown in Table 2. IDT website tools were utilized in selecting CRISPR-Cas12a guide oligos (crRNA). The PAM elements are not included in the synthesized RNA guides but are included in the table (bold) to demonstrate their importance for predicting specificity. gRNAs that are target conserved and possessed variable regions were purchased. The sequence of the reporter oligo is “56-FAM/TTATT/3IABkFQ” with a 6-carboxyfluorescein modification on the 5’ end and an Iowa Black™ fluorescence quencher modification on the 3’ end.

A standard 20 µL CRISPR detection reaction contains: ITD Alt-R® A.s Cas12a (cfp1) ultra [1.0–3.0 µL of a 1 µM solution] complexed to the complementary IDT crRNA [1.5–3.5 µL of a 1 µM solution]. The complex is incubated at room temperature for 10 min, then the IDT ssDNA green quench reporter [0.5–1 µL of 100 µM solution], NEBuffer™ r2.1 10× [2 µL], target DNA [1–5 µL (undiluted up to 1/100 dilution], and Ambion™ Nuclease Free Water (Invitrogen (Waltham, MA, USA)) are added to the reaction tube to the final volume of 20 µL [33]. The sequence of the addition of the reagents is important: Cas12a is complexed with crRNA for 10 min at room temperature, after which the remaining reagents are added and the reaction mixed by vortex prior to incubation at 37 °C for up to 30 min. The reaction demonstrated viability with a variety of targets, including phenol-extracted ethanol-purified DNA, 0.1% Triton X-100 crude parasite lysates, and RPA-amplified DNA.

### 2.5. Detection

For the qualitative assessment, the fluorescent signal of reaction tubes was observed visually after placement on a blue-light transilluminator (Invitrogen Safe Imager TM 2.0 Cat no. G6600) accompanied with an amber viewing cover. The transilluminator has a narrow emission peak centered at approximately 470 nm, which is compatible with the excitation wavelength of the fluorescent reporter tag in our diagnostic assay. Green fluorescent intensity was observed in relation to no-DNA control tubes. An inexpensive “blue light” flashlight will be included in future diagnostic kits for the excitation of the fluorescent signal.

For the quantitative fluorescent analysis during assay design, reactions were conducted in either 96-well (CellTreat; Ref: 229195) (80 µL reactions) or 384-well plates (Greiner (Kremsmunster, Austria): Ref: 781-096) (20 µL reactions) and placed into a fluorescent plate reader (Thermo Scientific (Waltham, MA, USA) Spectrophotometer Varioskan LUX machine, Ref# VLBL00D0) using an excitation wavelength of 485 nm and an emission of 520 nm. Unless indicated, the machine is pre-warmed to 37 °C prior to plate insertion and either single or kinetic loop readings are taken. The resulting information is exported from the SkanIT™ Software RE (ver. 7.0.0.50) into Excel for graphical production.

## 3. Results

### 3.1. Summary

**Figure 1:**
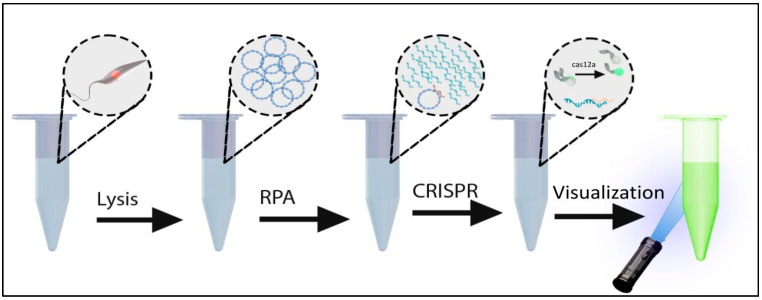
Diagram depicting the diagnostic assay incorporating Lysis, RPA, and CRISPR fluorescent detection.

A systematic approach was taken to develop an instrument-free Leishmania diagnostic. PCR-based diagnostics targeting kDNA are among the most sensitive methods for the detection of Leishmania parasites and are superior to microscopic methods in both sensitivity and specificity. Kinetoplasts are present in 30–50 copies per parasite and contain sequences that are both conserved and variable between species. Because PCR requires instrumentation and electricity, we selected isothermal RPA as a viable substation method for amplification, to both detect and amplify specific targets prior to the detection with Cas12a. To introduce additional specificity and sensitivity, we used CRISPR/Cas12a to detect the RPA amplicons. An overview of the process is depicted in Figure 1.

### 3.2. Identification of Target Sequence for Detection

Kinetoplast DNA (kDNA) forms an ultra-structure, specific to the genera of the family Trypanosoimatidae, that consists of a network of interlocking rings [40,41,42]. The unique kDNA structure was vaguely described after viewing under a phase contract by the renowned parasitologist William Trager in 1953 and continued to be illuminated in greater detail through advances in microscopic technology [43,44]. The kDNA target for conventional or quantitative PCR assays is composed of minicircle and maxicircle DNA molecules of unique kDNA [45]. When viewed under electron microscopy, the kDNA appears tightly packed in a disk-like structure [46,47]. For this diagnostic, we evaluated mini-circles, which are often the first choice for diagnostics because of their high copy number (10,000–20,000 copies/parasites). However, the high variability between Leishmania species, and even amongst strains of individual species, created challenges for the design of useful primers and probes [48]. Although parasites contain only 30–50 copies of maxicircles, they are more conserved within a species while exhibiting sufficient variation between species for diagnostic differentiation [49]. The robust amplification due to RPA more than compensates for the reduction in copy number to 30–50 per parasite.

### 3.3. Design and Testing of RPA Primers

The initial diagnostic is intended to comprise three channels for the detection of: (1) all Leishmania species, (2) species that cause mucosal disease, and (3) a human gene as a positive control. Later versions of the diagnostic will selectively detect *L. (L.) donovani* and *L. (L.) infantum* species that cause visceral disease in Old World countries. A 960 bp region from nucleotides 1808 to 2769 in the *Leishmania panamensis* strain PSC1 (maxicircle) kinetoplast (BK010875) was selected for amplification and detection due to the presence of sequences that are both conserved and variable between species. After the selection of maxicircles, a comprehensive analysis for the design of amplification primers and CRISPR guide sequences was performed. Analyses included ClustalW pairwise analysis of Genbank-deposited sequences covering all the major species available, including those that are associated with cutaneous or mucosal disease. Target regions for the first channel were selected for conservation amongst all Leishmania species but exclude sequences from the non-Leishmania Trypanosomatidae. In the second channel, only regions specific to mucocutaneous-associated species will be detected. In this paper, we focus on the specific detection of *L. (V.) panamensis*.

**Figure 2:**
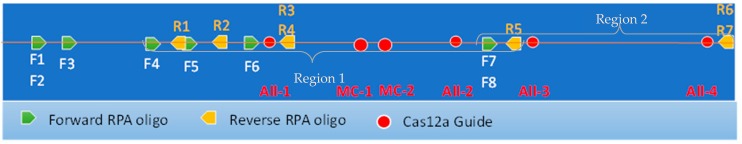
Map of 900bp target region of Leishmania maxicircle showing relative locations of all RPA oligos and CRISPR guides designed.

Eight forward and seven reverse oligonucleotide primers of 26 nucleotides were designed to amplify regions of kDNA containing both conserved and variable regions. Figure 2 shows the position of the “forward” and “reverse” primers within a 960 bp region of the maxicircle kinetoplast. The primers were combined as 24 pairs for the amplification of the two conserved regions that encompass the CRISPR guides All-1, -2, -3, and -4 (Table 1 and Figure 2).

### 3.4. Selection of RPA Primer Pairs

**Figure 3:**
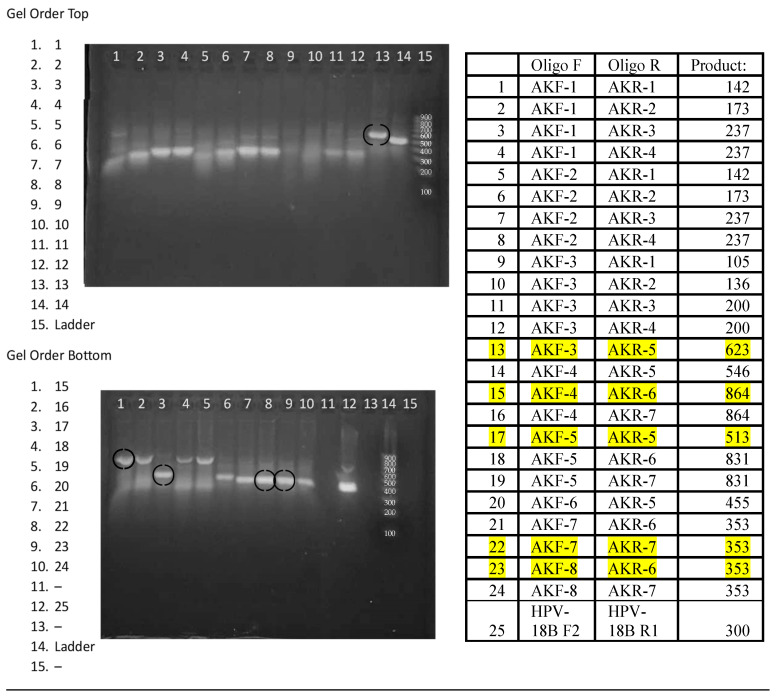
Agarose gel image demonstrating 24 primer pair RPA reactions with Leishmania parasite DNA.

**Figure 4:**
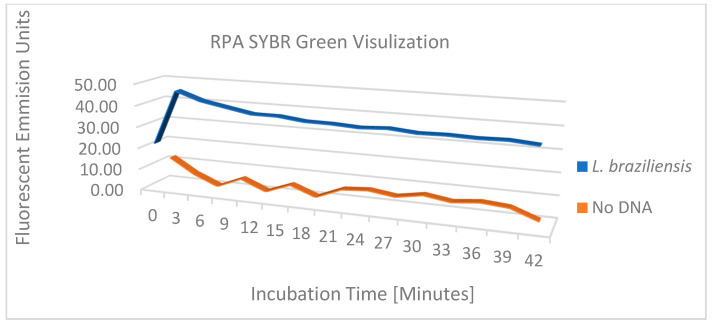
RPA amplification with SYBR Green for visualization of fluorescent output between *L. (V.) braziliensis* and no-DNA.

The performance of the 24 Leishmania primer pairs in RPA was assessed using DNA that was phenol-chloroform extracted from a pool of 3 × 10^6^ parasites equally composed of *L. (V.) panamensis, L. (V.) braziliensis*, and *L. (L.) infantum*, ethanol precipitated, and then resuspended in 0.45 mL of 20 mM Tris pH 7.4. Figure 3 shows the amplicons generated from 20 µL isothermal reactions, incubated for 20 min at 39 °C using ~2 × 10^4^ copies of purified DNA. While most reactions generated amplicons, five primer pairs were selected for continued analysis due to enhanced performance. Primer pairs #13 F3/R5 and #17 F5/R5 amplify region 1, primer pairs #22 F7/R7 and #23 F8/R6 amplify region 2, and primer pair #15 F4/R6 amplifies both regions 1 and 2 (Figure 2). To examine the amplification over time, the progress of the RPA reaction of *L. (V.) braziliensis* was monitored on a VarioSkan fluorescent plate reader with 50× SYBR Green and primer pair #15 F4/R6 and compared to the RPA reaction without target DNA (Figure 4). We observed a rapid increase in fluorescence, which indicates that the amplification of this reaction is rapid with a stable signal when compared to the negative control.

### 3.5. Parasite Lysis for RPA

**Figure 5:**
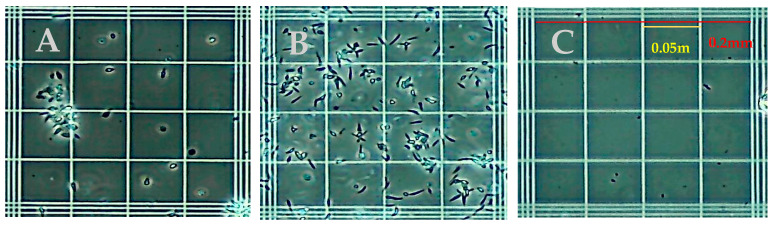
Phase contrast microscope images showing *L. (V.) panamensis* parasites (**A**) before lysis with grouping (**B**) before lysis vortexed and (**C**) after 0.1% Triton X-100 lysis.

Common detergent-based lysis buffers were assessed for the release of Leishmania DNA and compatibility with RPA. Initial studies assessed isotonic phosphate buffers containing CHAPS, Triton X-100, NP40, Tween-20, OCG at concentrations of 3%, 1%, 0.5%, and 0.1% (*v*/*v*) for the disruption of parasites and amplification by RPA. Microscopic inspection identified the minimum concentrations of each detergent that lysed the parasites. For each parasite tested, 0.1% (*v*/*v*) was sufficient to lyse parasites to an undetectable level in each microscope field examined. Figure 5 shows the appearance of native *L. (V.) panamensis* under phase-contrast microscopy on a standard hemocytometer, with Panel 5A demonstrating the preference of this species for aggregation, and Panel 5B demonstrating the dispersion slightly after vortexing. Panel 5C demonstrates *L. (V.) panamensis* parasites completely disrupted post lysis with 0.1% Triton X-100 detergent. Due to Triton X-100 detergents’ ability to fully lyse parasites, when compared to other reagents, and not interfere with the RPA amplification reaction, 0.1% of Triton X-100 was chosen to move forward in the assay development process.

### 3.6. Threshold of RPA Amplification

**Figure 6:**
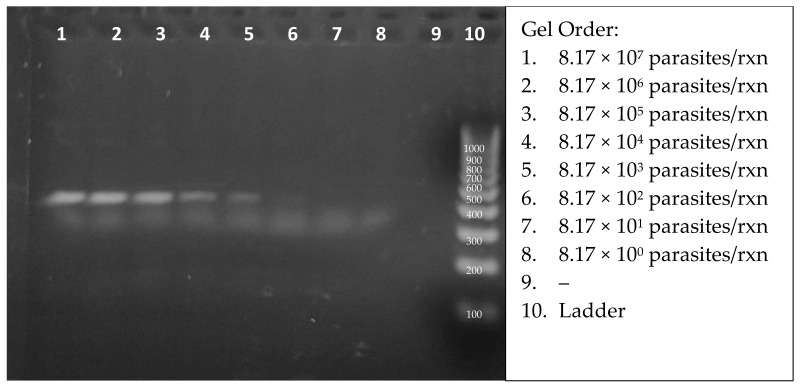
Agarose gel analysis of RPA-amplified products from 10-fold dilutions of starting lysed Leishmania DNA concentrations.

The viability of this diagnostic will be directly correlated to the sensitivity and specificity of detection. Therefore, the minimum parasite concentration was quantitatively determined in the RPA amplification assay. The ultimate goal would be to have the specificity above 97% and sensitivity under 10 parasites per complete RPA + CRISPR reaction. The number of parasites in a single infected macrophage is around this number [50]. The majority of the sensitivity will come from RPA amplification, but CRISPR will aid the process as well. Following protocol RPA conditions, with incubation at 39 °C for 20 min, a sample of lysed *L. (L.) venezuelensis* DNA at a concentration of 3 × 10^7^ parasites per mL was serially diluted for the assessment of the sensitivity of detection. Figure 6 shows the amplicons from 2.75 µL aliquots of 20 µL reactions. A careful inspection of the SYBR green-stained gel enabled the visual detection of target DNA from 817 parasites (lane 6). The true RPA threshold is likely below 817 parasites but is limited by the visual parameters of the gel. The quantifiable threshold of detection will be the combined RPA + CRISPR reaction, with more sensitive fluorescent emissions.

### 3.7. Variation of Time and Temperature for RPA

**Figure 7:**
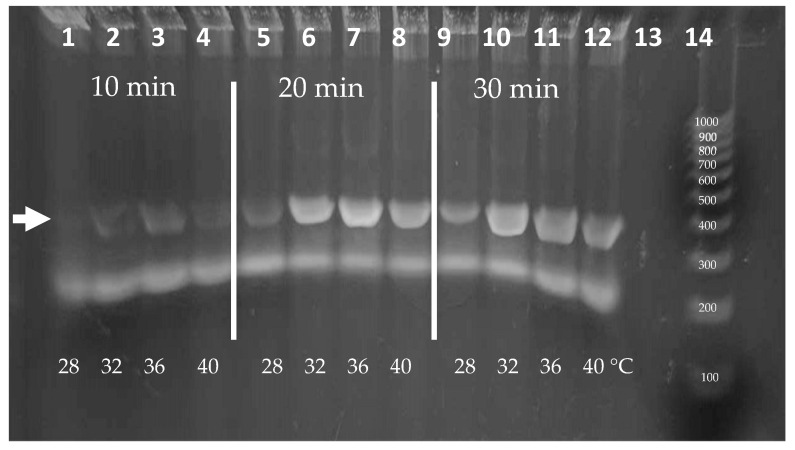
Agarose gel analysis of RPA using range of temperature and timepoint parameters. Arrow demonstrates target band size.

Isothermal amplification over a range of temperatures is advantageous for ease of use. RPA is known to have peak polymerase activity at parameters of 39 °C with typical reaction times of 20 min. Because ambient temperatures in areas where the diagnostic is intended to be deployed can vary, the effect of temperature on RPA performance was analyzed. We evaluated the parameters of incubation duration and temperature in strategic stepwise increments. To ensure that there was ample signal for visualization, experiments were conducted using 0.1% Triton X-100 lysates containing 2000 *L. (V.) panamensis* parasites per reaction. Figure 7 shows the amplicons produced following incubations of 10, 20, or 30 min at temperatures of 28, 32, 36, or 40 °C. Amplicons are visible at each temperature after a 20 min incubation. Additionally, while less vibrant, weak bands were visible after only 10 min. We note that additional sensitivity is produced during the following CRISPR detection step and that the amount of amplification to produce a visible band in an agarose gel (~25 ng or >108-fold amplification) may not be necessary. In the event that the ambient temperature is below 28 °C, the reactions could be heated using an inexpensive chemical hand warmer or a small battery-operated thermal chamber.

### 3.8. Two-Step Integrated CRISPR Detection

**Figure 8:**
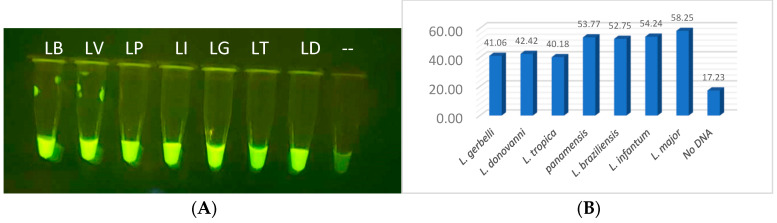
CRISPR analysis for all Leishmania visualized (**A**) in PCR tubes on blue light, (**B**) bar graphed based on quantitative fluorescent output from VarioSkan LUX machine.

The compatibility of the crRNA guide sequences to the RPA-amplified target DNA is critical to the CRISPR reaction, and the success of the diagnostic relies on the specificity from the CRISPR guide sensing. Without RPA amplification, the number of targets that can be detected with CRISPR alone is approximately 1000 to 2000, which is well above a usable field diagnostic for Leishmania. To properly evaluate CRISPR detection, purified RPA-amplified DNA was utilized to minimize incompatibility variables that might be found from inhibitors from crude parasites, the RPA reagents, and the CRISPR reagents. Correctly sized RPA DNA bands were gel-purified, ethanol precipitated and rehydrated with nuclease free water. The amplicon concentrations were determined by absorbance at 260 nm and the samples were normalized to 0.1 mg/µL. The Cas12a reactions were performed at 39 °C for 20 min, and probe cleavage emission was visualized on a blue-light transilluminator. All reactions produced visible fluorescent signals which glowed more intensely than the no-DNA negative control tube (Figure 8A). These results were confirmed using lysed parasites and RPA amplification with and without purification. Quantitative analysis was then performed using fragment DNAs with the VarioSkan fluorescent plate reader (Figure 8B).

### 3.9. Two-Step Integrated RPA–CRISPR Optimization

**Figure 9:**
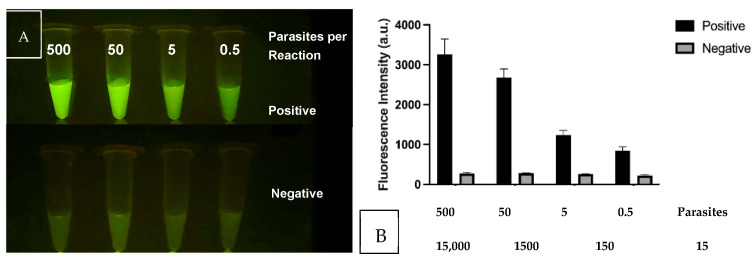
RPA–CRISPR threshold of detection experiment when 500, 50, 5 and 0.5 parasites were utilized in the RPA reaction whose product was then used with the corresponding CRISPR assay. (**A**) Fluorescent tubes visualization (**B**) Graph of Fluorescent intensity.

After confirmation that the CRISPR reactions detected their intended target sequences, we analyzed the threshold of detection in comparison to the background signal. Figure 9A shows the CRISPR/Cas12a signals as the DNA in the RPA–CRISPR reaction is reduced from 500 to 0.5 parasites. The negative reactions contained the CRISPR reagents without the inclusion of the RPA products. Both the specificity and sensitivity are enhanced through the combination of RPA + CRISPR than in either standalone reaction. The results also support the use of the multi-copy nature of the kDNA target as the tube containing 0.5 parasites produces a signal over the background. The lower set of tubes emulate negative samples and lack parasite DNA. Figure 9B shows the same samples analyzed for fluorescent intensity in the plate reader. In this study, the sensitivity was determined to be 0.5 lysed parasites per reaction or 15–25 kDNA targets, which is below our initial goal of 10 parasites per test.

**Figure 10:**
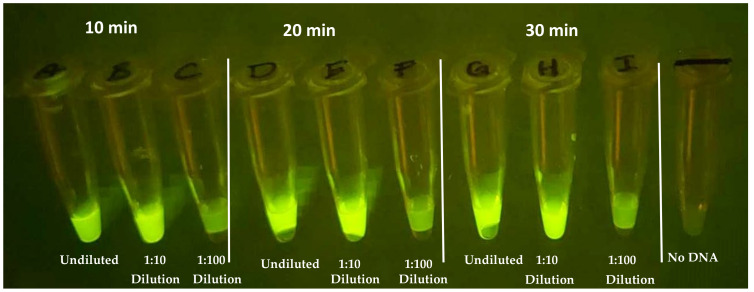
CRISPR reaction evaluating viability of RPA at three timepoints, 10, 20, 30 min, and three dilutions, undiluted, 1/10 and 1/100, alongside no-DNA control.

The evaluation of the incubation time and RPA product dilution for the RPA–CRISPR transition is critical for the development for compatibility with the device. Shorter assay times should translate into better field adoption, provided that the results remain consistent. The greater the dilution from RPA, the smaller the reaction volume can be yet still provide sufficient product for the CRISPR assay. RPA reactions were performed for 10, 20, and 30 min in 20 µL volumes to determine the impact that RPA incubation has on the CRISPR fluorescent signal. After the RPA step, 5 µL of the products, diluted and undiluted, were added to CRISPR reactions using guide All-3 with a 20 min incubation at 37 °C. Figure 10 demonstrates that, while not as vibrant as 20- and 30-minute incubation, as little as 10 min of RPA amplification produces a distinguishable fluorescent signal. In addition, undiluted and 1/10 dilutions of the RPA product had comparable signals; however, 1/100 was too diluted, in this experiment, for the current reaction parameters.

**Figure 11:**
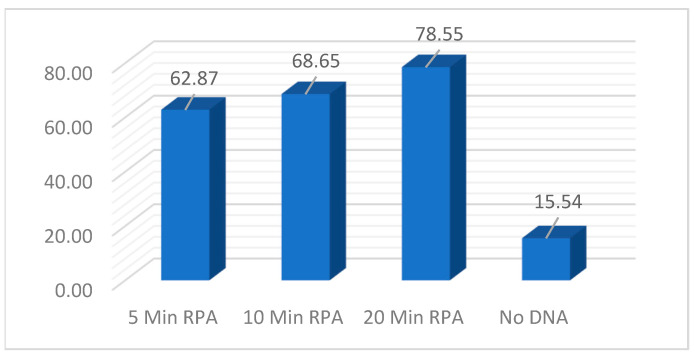
CRISPR reaction evaluating RPA incubation timepoints of 5, 10, and 20 min with a 1/10 of RPA product alongside a no-DNA control.

To further examine the impact that RPA amplification incubation time has on the CRISPR fluorescent signal, a timepoint experiment was performed. Identical reactions using 600 ng of DNA were utilized and timepoints of 5, 10, and 20 min were evaluated at 37 °C. Promptly, at the designated timepoints, 1/10 dilutions were made. In the CRISPR reaction, 5 uL of the 1/10 dilutions were utilized in the 384-well plates and the fluorescent signal was evaluated after 25 min. Figure 11 demonstrates that as little as 5 min of RPA amplification was enough to generate a strong fluorescent signal above the no-DNA background. While increasing the incubation does translate to a slightly stronger signal, additional experiments will be required to determine the statistical significance.

**Figure 12:**
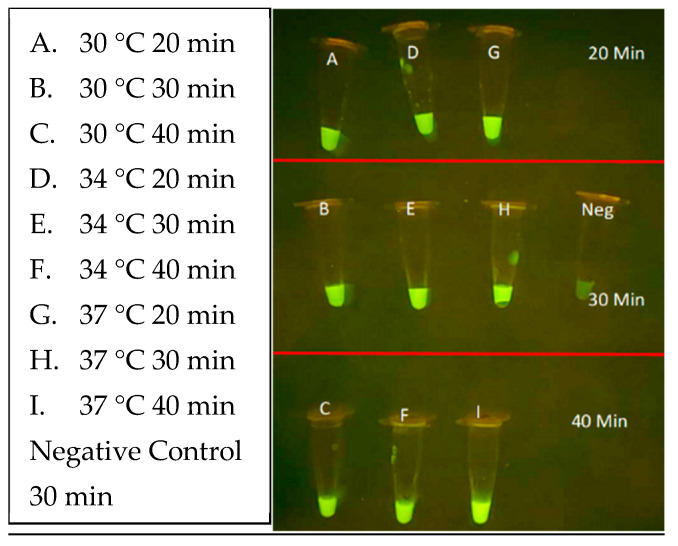
CRISPR optimization reaction evaluating time and temperature parameters of 30, 34 and 37 °C and incubation time of 20, 30 and 40 min alongside a no-DNA control.

Next, the CRISPR parameters of incubation duration and temperature were evaluated using unpurified *L. (L.) venezueliensis* lysates containing 2000 parasites per reaction. As shown in Figure 12, minimal differences are distinguishable between 20, 30, and 40 min reactions performed at 30, 34, and 37 °C. Shorter reaction times and wider ranges of temperatures are currently under evaluation.

### 3.10. CRISPR Specificity

**Figure 13:**
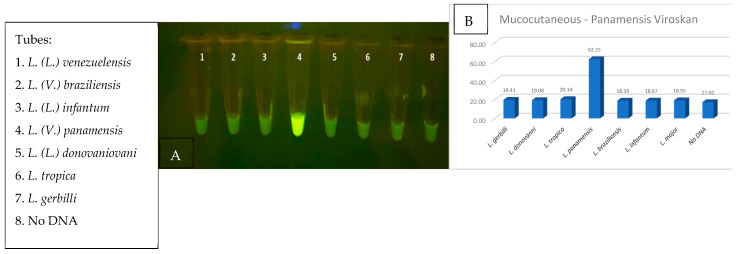
(**A**,**B**) RPA + CRISPR experiment demonstrating that only MCL-specific CRISPR was able to positively detect the *L. (V.) panamensis* DNA sample when identical reaction mixture was tested on all eight Leishmania parasite species.

Clinical providers in endemic countries highlight that differentiation between species of Leishmania parasites is an important component in the diagnosis and treatment of Leishmaniasis [30,50]. *L. (V.) panamensis* is a major contributor of mucocutaneous disease in Central America and was selected as the first target for specificity testing [13,51]. The objective was to locate sections unique only to *L. (V.) panamensis* species that share conservation to most *L. (V.) panamensis* and *L. (V.) guyanensis* isolates that could be encountered. After rounds of thorough multisequence alignment analysis, two promising sections of conservation were selected for the design of MC-1 and MC-2 CRISPR guides, located in region 1 of the maxicircle kinetoplast shown in Figure 2. Specificity was demonstrated when the fluorescent signal was restricted only to *L. (V.) panamensis* in comparison to the seven other Leishmania species, using either RPA-amplified lysed parasites or purified DNA fragments (Figure 13A,B, respectively). These results were upheld when the RPA amplification region was extended to span both regions 1 and 2 that would be required for multiplexing RPA amplification in the FAST device. Thus, the current design of the diagnostic is for Channel 1 to detect all Leishmania species and Channel 2 to detect species that progress to mucocutaneous disease. Future specificity testing will incorporate the mucocutaneous Leishmania species, *L. (V.) braziliensis* and *L. (V.) guyanensis,* for detection of the species in additional regions and include the specific detection of VL-causing species.

## 4. Discussion

We have demonstrated the viability of the individual assay components and shown how they can be performed in succession to detect single-digit Leishmania parasites for a field point-of-care diagnostic. The use of RPA to amplify target sequences and CRISPR to detect the amplicons increases both the specificity and sensitivity of the assay. Additional studies are planned to translate this proof-of-concept work into a valuable, working diagnostic tool.

As lysis and sample preparation can be inhibitory to diagnostic development, we sought to evaluate these parameters for incompatibility issues in succession to lysis performance. Of the many lysis reagents that are available, Triton X-100 was chosen. Our criteria required sufficient lysis for DNA release while not inhibiting the RPA amplification reaction at a final concentration of 0.1%. Additionally, as this diagnostic is intended for use in low-and-middle income countries, the price and availability of the reagent is also a priority. Triton X-100 can be obtained from many sources and is inexpensive. Our lysis conditions are compatible with all downstream reactions; however, these conditions are most comparable in the real world to test cell-free parasites from skin lesion swabs or scrapings. It will be important to test for inhibitors in these samples and also confirm lysis and the detection of amastigotes from infected macrophages.

The robustness of the RPA reaction and its performance over a range of times (5–30 min) and temperatures (28–40 °C) is a vital component in creating a standalone field diagnostic not beholden to modern diagnostic instrumentation. The robust isothermal reaction amplifies minute amounts of Leishmania DNA that can be visualized on agarose gels (approximately 20 ng) and detected in CRISPR reactions. Differences observed in the tubes will be evaluated in the devices to determine which parameters provide the clearest distinction between positive and negative results. In addition to the sensitivity, the specificity of the RPA primers is the first of a two-part system which allows for the detection of single species of Leishmania parasites required to identify the causative agent and triaging low verse high-risk prognosis. While all of the RPA primer combinations appeared to generate visible bands, a clear distinction was made for those which were advantageous over the others to move forward for the amplification of the desired regions.

**Figure 14:**
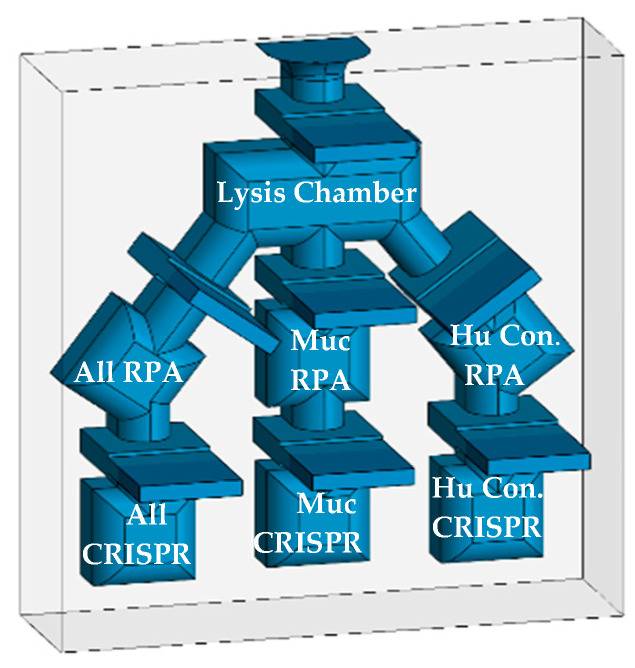
3-D depiction of the three-track diagnostic prototype with separate RPA and CRISPR chambers.

The specificity of the diagnostic was further enhanced by the use of CRISPR/Cas12a detection. The specific detection of *L. (V.) panamensis* among the field of all seven parasite strains was a remarkable result and was essential to achieving the required level of disease specificity. As with the RPA reaction, the CRISPR detection step demonstrated compatibility at a range of temperatures (30 to 37 °C), incubation durations (5–30 min) and up to 1/100 dilution of RPA-amplified DNA product. The visual detection of the green fluorescent signal was qualitatively and quantitatively evaluated, demonstrating clear distinctions between positive, negative, and control samples. We predict that the readout will be easily seen in the FAST device, which is transparent in the emission range. The FAST device shall be utilized in POC applications, when access to PCR diagnostics is prohibitive. Experimentation is in progress for the evaluation and optimization of the assay parameters in the FAST device to ensure that all aspects perform similarly as in tube or plate reactions (Figure 14). We are exploring options for the lyophilization of RPA and CRISPR reagents, which could be formed into pellets and introduced into each chamber of the diagnostic before backplate is attached. We also plan to assess multiplexing the three RPA reactions in one tube and develop methods to detect parasites in blood samples for the diagnosis of visceral disease. As mentioned previously, the fluorescent signal in the device will be evaluated in a variety of environments and a simple viewing chamber may facilitate detection in broad sunlight. Future studies will utilize de-identified patient samples for validation. We are also exploring other options for the FAST device system to include other pathogens (Malaria and HPV) as well as electrochemical sensors.

## 5. Conclusions

We have demonstrated the ability to detect single-digit parasites, without compromising the specificity in identifying single species, as proof of concept for a point-of-care diagnostic. In-tube analyses of individual assays were carried out in succession, culminating in an un-quenched fluorescent signal quantifiable over negative control. The described work is the foundation which will be implemented into a three-track [all Leishmania, mucocutaneous or visceral only, and a human positive control] assay that we plan to utilize in a Funnel Adapted Sensing Tube (FAST) single use, instrument free, and affordable diagnostic.

## 6. Patents

US20240085406A1: priority date of September 2022. “Multi-chamber device for detecting pathogens/molecules and methods of using same”.

## Figures and Tables

**Table 1:** Details for the eight forward and seven reverse RPA oligos designed for the amplification of region 1 and/or region 2 on the maxicircle kinetoplast.

Primer Name	Nucleotide Sequence	Length	Nt. Location in PSC1 Kinetoplast ACC#BK010875
F1	GGCAAGTCCTACTCTCCTTTACAAAG	26	1808..1838
F2	GGCAAGTCCTACTCTCCTTTACAAAGAGAAC	31	1808..1833
F3	TTGTATGTTTGATTGGGGCAATACT	25	1851..1875
F4	AGGTTCGAGCAGGTTAACAAGC	22	1922..1943
F5	ATGTGTTTCATCGTCTACTTATTGC	25	1955..1979
F6	TTCGTTAGTTGGGTTAAAATCGTTG	25	2012..2036
F7	GATGCCAGCCGTTGCGGTAATTTCTATGC	29	2417..2445
F8	GATGCCAGCCGTTGCGGTAATTTC	24	2417..2440
R1	ATTAATGCTTGTTAACCTGCTCGAAC	26	rev:1924..1949
R2	TAGCAATAAGTAGACGATGAAACAC	25	rev:1957..1981
R3	TGCTTTACAACGATTTTAACCCAAC	25	rev:2019..2043
R4	TGCTTTACAACGATTTTAACCCAACTAACG	30	rev:2014..2043
R5	TAAAAGCATAGAAATTACCGCAACG	25	rev:2426..2450
R6	GTTGTCTTTATTACAAAGAATGGTGGGCAAC	31	rev:2738..2768
R7	GTTGTCTTTATTACAAAGAATGGTG	25	rev:2744..2768

**Table 2:** Details for the six CRISPR guides designed for the detection of all Leishmania and mucocutaneous specific species. PAM sequence in bold is not included in the oligo.

Guide Name	Nucleotide Target Sequence	Length	Location on Maxicircle
All-1	**TTTA**CAACGATTTTAACCCAACTAA	25 (21 + PAM)	rev:2016..2040
All-2	**TTTA**GGAATAGTTAATAATAATTTA	25 (21 + PAM)	2284..2308
All-3	**TTTG**ACAACATGATAAGGATTATAA	25 (21 + PAM)	2629..2653
All-4	**TTTA**TAAAATAAATGTATAATATTT	25 (21 + PAM)	rev:2450..2474
MC-1	***TTTA***AAAATATAAAAGTCAATTGTT	25 (21 + PAM)	2138..2161
MC-2	***TTTA***TATTATTTTATATTATTTTAT	25 (21 + PAM)	2178..2201

## Data Availability

The original contributions presented in this study are included in the article. Further inquiries can be directed to the corresponding author.
